# Heat Synchronization Protocols in Dairy Cattle (*Bos indicus*) and Success Factors in Niger

**DOI:** 10.1155/vmi/4212869

**Published:** 2026-08-17

**Authors:** Mahamadou Moussa Garba, Mani Mamman, Hamadou Issa

**Affiliations:** ^1^ Department of Animal Production, National Institute of Agricultural Research of Niger, P.O. Box 429, Niamey, Niger

**Keywords:** artificial insemination, estrus synchronization, intravaginal devices

## Abstract

Synchronization and induction of ovulation enable the control and harmonization of femaleestrus cycle facilitating artificial insemination (AI) by mitigating the challenges associated with heat detection. In Niger, progestogens alone or in combination with other hormones such asgonadotropin‐releasing hormone (GnRH) and prostaglandin F2alpha (PGF_2α_), are frequently employed to manage prolonged postpartum anestrus in cattle. This summary reviews their physiological mechanisms of action and practical application protocols. Additionally, complementary hormonal treatments notably PGF_2α_ and Equine chorionic gonadotropin (eCG) are commonly integrated intointravaginal device (IVD) based regimens. Understanding the hormonal dynamics of estrus is essential for maximizing the success rate of IVD‐based synchronization program.

## 1. Introduction

Livestock farming plays an important socioeconomic role in West Africa, accounting for between 5% and 44% of agriculture in most countries in the region. In addition, it plays a central role in (i) preserving and improving the environment [1]; (ii) food security, through its contribution of animal protein; and (iii) poverty reduction, through diversification and increased incomes for local populations [2]. However, the West African population’s demand for meat and dairy products remains largely unmet [3]. Extensive farming, which is widespread in West Africa, is unable to provide the meat and milk needed by the entire population concerned under satisfactory economic conditions. This shortfall in milk production is due to various political, climatic, and economic constraints, but also to the low milk yield of local breeds and extensive production methods on poor pastures, which are often only available seasonally. In addition, health and social constraints and the lack of technical support for farmers also hinder technological innovation. Infertility is a major constraint in Africa cattle breeding, leading to an increased age at first calving and prolonged calving intervals. Postpartum anestrus is the main factor that negatively affects the reproductive performance of animals bred in the tropics. The factors responsible for this infertility are diverse in nature. They concern the animal (age, parity, body condition, postpartum, or gestation pathologies) or its environment, namely, diet, heat stress, husbandry practices, and even hormonal protocols [4, 5].

For many years, researchers in tropical and subtropical countries have been using various methods to improve the fertility of cattle herds in order to meet the population’s needs for milk and dairy products. These methods include AI, which has been recognized as a primary tool enhancing cattle productivity. In sub‐Saharan Africa, it is deployed across various breeding programs aimed at enhancing meat and, most notably, milk production. Although performed during natural estrus, it is far commonly carried out following estrus synchronization [6]. Given the constraints associated with the manifestation and detection of estrus, it must most often be performed after hormonal induction using progesterone (P4) releasing IVD in particular PRID^ND^, controlled internal drug releasing (CIDR^ND^), combined or not, depending on the protocols, with PGF_2α_, eCG, and GnRH. These protocols enable the systematic insemination of a larger number of animals [7]. In doing so, they help to reduce infertility without necessarily improving fertility [5, 6, 8]. These protocols make it possible to dispense with heat observation and to systematically inseminate a larger number of animals [7]. However, fertility rates during induced estrus vary greatly between farms. Estrus synchronization and its various protocols are useful tools in a cow herd’s reproductive management, but they require optimal diet, good body condition, and health, as well as experienced manpower. A good understanding of the physiological mechanisms behind ovulation synchronization and, in some cases, induction, provides some insight into the limitations of currently available treatments.

Cattle farming in Niger is an important activity that plays a major role in the national economy and contributes to food security and rural incomes. The cattle population is estimated at over 11 million head. It consists of several local breeds belonging to the genus *Bos taurus* (Kouri), but mainly *Bos indicus* (Azawak, Bororo, Djelli, Goudali). Few studies have been devoted to the reproductive performance of African zebu cattle. The age at first calving and the calving interval range from 30 to 60 months and 14 to 16 months, respectively. These averages depend on the breeding conditions of the studies conducted. In Niger, these two parameters are estimated at 36.5 and 14 months, respectively, for farm‐raised Azawak cows. Given these performance levels, various hormonal protocols aimed at inducing and synchronizing ovulation have been evaluated to enable more intensive use of AI [9].

In Niger, before hormone treatments were introduced, the cyclical nature of females was most often checked by transrectal palpation or ultrasound. A study conducted at a station on female cattle treated with PGF_2α_ and progestogens made it possible to determine the exact time of AI by monitoring follicular growth until ovulation [10]. Generally, before insemination, the quality of the semen is examined in the laboratory to assess its motility and mass motility. Females were inseminated after ultrasound examination to confirm the disappearance of the largest follicle or a sudden drop in its diameter. Several AI following hormone treatment were carried out in Niger on female Azawak, Djelli, Goudali, Bororo, and Kouri cattle raised at the station or on private farms using fresh or frozen semen from Azawak bulls or frozen semen from European breeds, notably Piedmontese, Holstein, Brune des Alpes, and Valdôtaine [11]. However, the implementation of these development strategies and programs has not improved the performance of the livestock sector, and indicators, such as per capita meat and milk consumption, have fallen significantly. Yet Niger has local animal genetic resources which, if better exploited and managed, could significantly improve the performance of local breeds.

Several factors influence reproductive success following fixed‐time artificial insemination protocols in cattle [12, 13]. Some authors mention the use of new or reused IVD [14], hormonal treatments [15, 16], follicle size or diameter, and poor body condition score at estrus synchronization [17].

The objective of this study is to review estrus induction and ovulation synchronization protocols used during AI in Niger in *Bos indicus* dairy cattle in Niger, as well as the key factors influencing their success.

## 2. Reproductive Physiology in *Bos indicus* Cows

The estrus cycle in cattle typically ranges from 18–24 days (averaging 21 days). It consists of a luteal phase (14–18 days) and a follicular phase (4–6 days). During the cycle, there are generally two (dairy cows) or three (heifers and beef cows) waves of ovarian follicle growth. Each wave of follicle growth consists of a period of emergence of a cohort of follicles, selection of a dominant follicle, and either atresia or ovulation of the dominant follicle. These waves of follicle growth, initially established during the early pre‐pubertal period of development, occur throughout the entire cycle, with only the dominant follicle of the final wave coinciding with the follicular phase that undergoes final maturation and ovulation.

Compared to the *Bos taurus*, the zebu is characterized by

• Smaller genital tract dimensions (study carried out on 500 genital tracts of Azawak, Bororo, Djelli, and Goudali zebus taken at the slaughterhouse of Niamey) [8];
• Zebu heifers reach sexual maturity and puberty later than *Bos taurus* heifers. The estimated age at puberty for Zebu in the tropics and subtropics is between 16 and 40 months, with a mean of 25 months, i.e., 6 to 12 months later than *Bos taurus* [18, 19];
• *Bos indicus* presents reproductive behavior different from that of *Bos taurus*, with a short duration of estrus, and high rates of estrus expression during the night [20];
• The maximum diameter of the ovulatory follicle were, on average 14 to 17 mm in *Bos taurus* [21] and 11.3 to 12.3 mm in *Bos indicus cattle* [22];
• The maximum corpus luteum diameter was between 20 and 30 mm in *Bos taurus* and between 17 and 21 mm in *Bos indicus* [22] females;
• Many studies have reported that the number of ovarian antral follicles is greater in *Bos indicus* than in *Bos taurus* breeds [23];
• Bastos et al. [24] observed a greater number of small follicles (2–5 mm) in the ovaries of *Bos indicus* than *Bos taurus* cows at the time of wave emergence. This difference in the number of small follicles between those breeds persisted throughout the estrous cycle;
• A smaller diameter of the preovulatory dominant follicle (10 to 12 mm vs. 14 to 20 mm) and of the corpus luteum (17 to 21 mm vs. 20 to 30 mm); [25].

## 3. Hormonal Regulation of the Sexual Cycle

The hormonal regulation of the sexual cycle is very complex, involving not only the follicles, corpus luteum, and endometrium, but also the pituitary gland and hypothalamus. Each of these anatomical structures is responsible for hormone synthesis. The follicles synthesize estrogen and inhibin, the corpus luteum synthesizes P4 and oxytocin, the endometrium synthesizes PGF_2α_, the pituitary gland synthesizes luteinizing hormone (LH) and follicle‐stimulating hormone (FSH), and the hypothalamus synthesizes GnRH, thereby coordinating ovulation, luteolysis, and implantation. These hormones act through a system of positive and negative feedback to control the estrus cycle in cattle. The cycle consists of two distinct phases: the luteal phase (14 to 18 days) and the follicular phase (4 to 6 days). The luteal phase is the period following ovulation, during which the corpus luteum forms (often referred to as metestrus and diestrus). The follicular phase is the period following the disappearance of the corpus luteum (luteolysis) until ovulation (often referred to as proestrus and estrus). During the follicular phase, the ovulatory follicle reaches its final maturation and ovulation occurs; the oocyte is released into the oviduct, allowing for possible fertilization. Ovulation occurs 10 to 14 h after estrus and is followed by the luteal phase of the estrus cycle. After ovulation, P4 concentration increases due to the formation of the corpus luteum, in which the granulosa and theca cells of the dominant ovulated follicle luteinize and produce P4 for the establishment and maintenance of pregnancy and/or the resumption of the estrus cycle.

After estrus, during metestrus, the corpus luteum develops and P4 levels rise. Estradiol concentration falls during the first 48 h after estrus. This results in a progressive increase in FSH, responsible for the development of follicles larger than 4 mm in diameter during the first wave of follicular growth. These growing follicles synthesize both estradiol and inhibin. The combined action of these two hormones results in a reduction in FSH synthesis and is responsible for the progressive selection of a dominant follicle, with the surplus follicles atrescending. The terminal stage of the dominantphase involves a pronounced increase in estrogen secretion, independentof progesterone (P4)influence. However, if the dominant follicle is in a period of maximum P4 impregnation, this estradiol synthesis is not prolonged. Dominance ends, the follicle atresises and a new wave of follicular growth may appear, preceded by a further increase in FSH.

Data for hormone level parameters include 13 articles, resulting in 21 studies on P4, 8 studies on estrogen, 6 studies on FSH, and 8 studies on LH [20]. The meta‐analysis result indicates a variation in P4 levels during the proestrus phase and reproductive performance parameter between *Bos indicus* crossbred and their crossbred with *Bos taurus*; however, there was no difference in estrogen, FSH, and LH levels, as well as estrus activity parameter between the two species. The increase in P4 levels during proestrus phase on crossbred cattle is the reason for the occurrence of delayed ovulation, thus requiring external hormone intervention.

Data indicate that it is LH the limiting hormone for the onset of postpartum ovarian activity. After LH stores are re‐established, nutritional status and suckling may be the most important factors that inhibit postpartum ovulation [26]. Poor nutrition is a recognized cause of reduced fertility in cattle grazing in subtropical/tropical areas [25].

Monitoring circulating P4 concentrations during follicular development is a fundamental aspect of synchronization protocols for fixed‐time artificial insemination in cattle. Follicular growth is mainly controlled by the frequency of LH peaks, which is itself modulated by circulating steroid hormone concentrations, such as estradiol and P4 [27].

## 4. Hormone Treatments to Induce/Synchronize Ovulation in Zebu Cattle

Various hormonal treatments for synchronizing estrus and ovulation are available for cattle, using only hormones such as PGF_2α_ or in combination with other hormones such as P4, estrogen, and GnRH [28]. Progesterone‐impregnated intravaginal devices are: CIDR (1.38 g of P4, Zoetis), PRID Delta (1.55 g of P4, CEVA), and Cue‐Mate (1.56 g of P4, Vetoquinol). They are used in combination with PGF_2α_, eCG, and estrogens or with GnRH. These treatments need to induce a high and quick elevation of plasma P4 concentrations in the cow and should maintain this concentration above 2 ng/mL until removal [29]. Synchronization improves reproductive efficiency by reducing the interval between calving, planning the calving season, and effectively using artificial insemination techniques [30]. The main limitation of ovulation‐estrus synchronization programs is their inability to induce potentially fertile estrus and ovulation in noncyclical cattle [28].

PGF_2α_ is used to induce luteolysis in a cycled animal. Estrus synchronization using this product and/or its potent analog is the treatment of choice in the reproductive management of cattle [31]. Two recent publications have provided an overview of the research conducted over the past 100 years that has led to the identification of the luteolytic role of PGF_2α_ in cycle regulation and its applications in bovine reproduction [7, 32, 33]. This regulation initially involved a single or double injection of PGF_2α_ [34]. It was subsequently widely used in Ovsynch‐type protocols [35]. While the method is attractive, the fertility rate after estrus varies greatly between farms, but also within the same farm from one batch to another and from 1 year to another [36]. However, in zebu cattle, given the low percentage of animals observed in estrus due to the constraints associated with its detection, the injection of PGF_2α_ often results in a low pregnancy rate. But alternative treatments have been proposed for *Bos indicus*.

Ovsynch is one such method. It consists of an initial injection of GnRH (100 mcg intramusculary) to trigger the start of a new follicular wave within 3 or 4 days. This is followed by an injection of PGF_2α_ (25 mg) 7 days after the GnRH injection, which aims to lyse the primary and secondary corpus luteum (resulting from the GnRH injection). Finally, a second injection of GnRH is given 2 days after the PGF_2α_ injection to increase the accuracy of the dominant follicle’s ovulation period, AI 12 to 24 h after the last GnRH injection. The effects of GnRH on LH and FSH make it possible to use it (1) during estrus or at the time of insemination, (2) early (D4 to D9) or mid (D11 to D14) in diestrus, (3) during embryo transfer, (4) upon early confirmation of pregnancy (D30), (5) in cases of ovarian cysts, and (6) during the postpartum period to induce cyclic activity [37, 38]. Studies have assessed the effectiveness of the Ovsynch protocol in inducing estrus [6] and on reproductive performance [39].

There are also two progestogen‐based protocols for inducing/synchronizing estrus. These involve the IVD in Niger: CEVA’s PRID Delta and ZOETIS’s CIDR for 7 to 8 days. During implementation, an intramuscular injection of 5 mg (heifers) or 10 mg (cows) of estradiol benzoate is administered. This inhibits the growth of any dominant follicle that may be present and induces a new wave of follicular growth. When administered at the start of P4 therapy, estradiol esters (benzoate: 10 mg, cypionate: 5 mg) cause follicular regression due to their inhibitory effect on FSH [40, 41]. When the CIDR or PRID is removed, an injection of PGF_2α_ is given to luteolyse any corpus luteum that may be present. After 24 h, a second injection of estradiol benzoate (5 mg) is given. The purpose of this is to increase the degree of synchronization of estrus and ovulation and thus improve the pregnancy rate. Systematic insemination is performed 52 to 56 h after removal of the PRID or CIDR. If necessary, a GnRH injection may be administered simultaneously with AI to increase the ovulation rate.

The use of an IVD can be considered in two contexts: zootechnical or pathological. The first concerns farms practicing group calving, and the second concerns animals in pathological functional anestrus. In both cases, insemination can be performed after detection of estrus, or systematically after removal of the IVD.

The use of IVD involves a number of constraints:

• Animals must be immobilized several times, depending on the additional hormone treatments required;
• The policy of grouping calving;
• Compliance with hygiene rules during insertion;
• Consideration of the animals’ body condition (cad > 2.5);
• The costs of treatments and clinical examinations.

To better understand the rationale for complementary hormone treatments when using particularly PGF_2α_ and eCG, it should be noted that [29]

• P4 aims to reduce LH secretion and prevent the onset of estrus and ovulation. Removal of the IVD ensures the resumption of follicular growth. Reducing the duration of treatment from 3 to 5 days (7 to 9 days compared to 12 days previously) reduces the duration of follicular dominance and helps to increase fertility;
• PGF_2α_ injected 24 h before removal of the IVD induces luteolysis of any corpus luteum present (as well as that of the dominant follicle luteinized by GnRH injection at the start of treatment) and the lowest possible P4 concentration during estrus;
• eCG is much more commonly used in heat synchronization protocols based on prolonged‐release progestogen devices. In noncyclic animals, eCG is injected at a dose of 400 IU at the time of IVD removal to improve follicular growth and fertility [42];
• GnRH injection at the time of IVD insertion is intended to induce ovulation or luteinization of any dominant follicle present, as well as the onset of new follicular growth 2 to 3 days later;
• Synchronization is more effective in females that are in heat at the start of the synchronization treatment;
• Good detection of estrus: this determines the length of the waiting and breeding periods;
• Good breeding conditions: obstetric manipulations, poor hygiene, and uterine infections cause a delay in the resumption of postpartum cyclicity and poor fertility;
• Balanced diet: poor nutrition causes functional anestrus or pathological functional anestrus. It should be noted that LH concentration (important for optimal follicular growth) is directly proportional to energy intake.

Estrus synchronization protocols using IVD offer several advantages, including

• Reduction in the interval between calving;
• Reduction in age at first calving;
• Elimination of heat monitoring;
• Ability to inseminate a group of females at a specific time;
• Ability to group births;
• Ability to improve reproductive efficiency;
• In animals in anestrus, it is recommended to inject 300 to 500 IU of eCG when removing the PRID or CIDR. This treatment promotes the development of a dominant follicle.

Table 1 summarizes several protocols for ovulation synchronization in *Bos indicus* and the results obtained.

**TABLE 1:** Summary of some ovulation synchronization protocols in *Bos indicus*.

Protocol	Results obtained.	References
PRID^ND^, D8 injection of 25 mg of PGF_2α_ (Enzaprost), D10 Prid Withdrawal + 500 IU d’eCG, D12 AI	100% of synchronization rate; 61%, 59% of gestation rate; ↑ fertility with parity (44%, 89% vs 74%).	[43]

PRID^ND^, J6 injection of 25 mg of PGF_2α_ (Enzaprost), D7 Prid Withdrawal D9‐D10 AI	↑ synchronization rate with parity (90% vs 64%, 7%); ↑ gestation rate with parity (70% vs 64%.7%).	[44]

Group 1: GPG protocol in *Bos indicus* Group 2: CoSynch + progesterone: *Bos indicus* × *Bos taurus* Group 3: progesterone + eCG: *Bos taurus*	↑ the percentage of pregnancies *Bos indicus* × *Bos taurus* compared to *Bos indicus* (57%, 6% vs 41%, 1%); ↑ fertility and reproductive performance in stabled animals, respectively (60, 8 vs 46%, 5%) (111, 8 ± 41.6 vs 277, 4 ± 128 hours).	[45]

PRID, D8 injection of 10 mg PGF_2α_, D9 Prid Withdrawal + injection of 750 IU of PMSG, D11 TAI	No significant difference (*p* > 0.05) between the different BCS on the pregnancy rate; number of days elapsed from the last birth until the day of artificial insemination had no significant influence (*p* > 0.05) on the pregnancy rate; dietary supplementation had no significant effect (*p* > 0.05) on the success rate after AI	[46]

Group 1: injection of 125 mg PGF_2α_ (Dinoprost) Group 2: PRID, D8 Prid Withdrawal + injection of 125 mg of PGF_2α_ + 400 IU of eCG (Folligon) D9 1AI, D10 2AI Group 3: PRID, D8 Prid Withdrawal + 125 mg PGF_2α_ + 800 IU of eCG (Folligon) D9 1AI, D10 2AI	Average time between the injection of PGF_2α_ or PRID removal and the detection of the estrus appeared to be significantly longer (*p* = 0.01) among the females of group 1 (84.8 ± 26.0 h) than in those of groups 2 (60.3 ± 20.7 h) and 3 (56.2 ± 10.5 h); Average duration of the estrus was significantly shorter (*p* 0.0001) among the females of group 1 (12.6 ± 2.6 h) than among those of groups 2 (22.6 ± 2.8 h) or 3 (23.7 ± 2.5 h); Interval between treatment and ovulation was significantly longer (*P* = 0.006) for the animals of group 1 (112.8 ± 4.9 h) than for those of groups 2 (88.4 ± 9.5 h) and 3 (85.5 ± 13.7 h).	[10]

Ovsynch group: (100 μg of cystorelin + 500 μg of cloprostenol + 100 μg of cystorelin Control group: AI during the natural estrus cycle	The pregnancy rates is higher in Ovsynch group with overall 62.5% and 50% in the control group but no statistically significant difference; The estrus response was significantly higher in the control group (54.5% vs 29.17%); The estrus return was significantly higher in the Ovsynch group compared with the control group (33.3% vs 18.2%).	[39]

Ovsynch (Buserelin acetate) followed by 500 μg Cloprostenol + GnRH 48 h after PGF_2α_ administration and after 18 to 24 h of second GnRH administration FTAI was done	No breed variations were found for the estrous induction rate (89.47%) and (75.0%) in repeat breeding and postpartum anestrous cows, respectively; No significant differences were observed for the average time required for onset of estrus between the two types of reproductive disorders; No significant differences about average duration of estrous (h) and conception rate (%)	[47]

CoSynch standard vs. CoSynch + P4	Adding P4 (intravaginal device) to the standard protocol significantly improves fertility, achieving satisfactory rates of 80% in cows that have come into heat, compared to 35% with CoSynch + P4 and only 10.5% with standard CoSynch	[6]

Single injection of PGF_2α_ Two injections of PGF_2α_ 11 days apart	A single injection is more cost‐effective; it effectively induces estrus, often followed by artificial insemination (AI) during observed estrus; Double injection is particularly effective for synchronizing estrus, allowing for FT AI (80 h after the second injection)	[36]

Group 1: Norgestomet combination/estradiol/PGF_2α_/PMSG, AI 48^th^ and 72^nd^ Group 2: 2 injections PGF_2α_ a 11jours d’intervalle, AI 72^nd^ and 96^th^ h after the second injection of PGF_2α_	The hormone combination achieved a higher pregnancy rate of 66.66% (excluding implant losses), while PGF_2α_ alone yielded a lower 29.41% fertility rate.	[48]

Double injection PGF_2α_ (10 mg Cloprostenol) Single injection PGF_2α_ 10 mg alfagladin C (Cloprostenol)	Double injection of PGF_2α_ estrus synchronization protocol (100% response) was more effective than a single injection (90.8% response) in dairy cows, with an overall conception rate of 48.3%. The double injection method showed higher efficiency in both estrus induction and pregnancy rates	[49]

Group 1: CIDR+25 mg PGF_2α_ and 0.5 mg Estradiol cypionate Group 2: CIDR+25 mg PGF_2α_ and 24 h later (day 8), 1 mg Estradiol benzoate	The overall estrous rate was 73.32%; Estradiol cypionate had a higher rate (79.39%) compared to Estradiol benzoate (67.19%);	[50]

*Note:* ↑, increase; D, day; GPG, gonadropins‐PGF_2α_‐gonadotropins; h, hours.

Abbreviations: 1AI, first artificial insemination; 2AI, second artificial insemination; AI, artificial insemination; BCS, body condition score; FTAI, fixed time artificial insemination; IU, international unity; TAI, time artificial insemination.

The main synchronization methods used in Niger are as follows:

• Prostaglandins to induce luteolysis. These are effective on cows in heat, often in two injections spaced apart, to synchronize ovulation in 2 to 5 days.
• P4 and IVD (CIDR/PRID): Insertion of a device containing P4 for 7 to 9 days, followed by an injection of prostaglandins/eCG upon removal. This is commonly used in cows in anestrus (not cycling).
• GnRH‐based protocols (Ovsynch/Cosynch): Combine GnRH and PGF_2α_ to enable fixed‐time insemination, thus eliminating the need for heat detection, especially in the context of extensive farming in Niger.
• Hormone combinations (P4 + estrogens + eCG) which are effective for cows in anestrus or for optimizing results.

A summary of 1348 treatments performed on *Bos indicus*, mainly of the Azawak or Nellore breeds, shows that (1) estrus occurs on average 28.3 h (15 to 35.9 h) after withdrawal of the progestogen, (2) the average rate of observed heat is 82.1% (57.1% to 100%), (3) the average duration of heat is 11.8 h (11.3 to 12.2 h), and (4) the average pregnancy rate is 46.6% (22% to 57.6%) [9]. The disparity in results observed between studies is attributable to a variety of hormonal and zootechnical factors. The stage of the animal’s cycle at which treatment is carried out has an effect on the response time, degree of synchronization, and pregnancy rate.

Unfortunately, very little of this work has been published and is most often the subject of student theses. According to insemination results from 2001 to 2010, female responses to heat induction varied depending on the protocol (higher response with Crestar) and the season (reduced expression of estrus during the rainy season). AI following the use of heat synchronization protocols results in low pregnancy rates (29%–30%). However, fertility after insemination during natural heat was significantly higher than after induced heat (43.4% vs. 25.6%). Also, fertility after insemination with fresh semen is higher than that achieved with frozen semen (31.2% vs. 21.7%) [9].

Several factors including hormonal, therapeutic, and herd managementpractices can affect fertility and fecundity after progestogen induction or synchronization. These include hormonal, therapeutic, and zootechnical factors.

Analysis of the situation in Niger suggests that: (1) good health monitoring is required for hormone management, (2) synchronization with fixed‐time AI would maximize success rates due to heat and nutritional stress, and (3) P4 devices are preferred for managing frequent anestrus in local zebu cattle.

## 5. Conclusion

Heat synchronization is useful for managing reproduction in a herd of cows. However, it requires sufficient feed and good livestock health to achieve the best results. Several therapeutic treatments have been applied in *Bos indicus*, yielding satisfactory results in terms of fertility. Progestogens are therefore a means of optimizing bovine reproduction management.

The results from Niger confirm that artificial insemination is a viable tool for genetic improvement in cattle. However, optimizing this technique requires addressing various influencing. These include the importance of clinical examination of animals before starting hormone treatment to induce heat. This examination would allow for better selection of animals based on their weight, body condition, and whether or not they are in heat. It would also be important to improve the conditions for using frozen semen. From a zootechnical standpoint, it would undoubtedly be essential to analyze possible avenues for improvement in heat detection and feeding. In practical terms, these insights pave the way formore reliable field protocols that maximize response rates while optimizingresource use. Adopting a holistic approach combining rigorous animal selection,improved semen handling, and better herd management will ensure the practicalsuccess and long‐term viability of cattle genetic improvement programs.

## Funding

This work did not receive any funding.

## Conflicts of Interest

The authors declare no conflicts of interest.

## Data Availability

The data that support the findings of this study are openly available in article de synthèse at https://submission.wiley.com/submission/submissionBoard/de14d8ed-a68d-472c-a449-a67486e58187.
